# Vaginal Birth at 8 cm Because of Severe Cervical Oedema: A Case Report

**DOI:** 10.1155/crog/1085385

**Published:** 2026-07-27

**Authors:** Pécheux Océane, Patet Emma, Crofts Victoria, McCarey Catherine, Pellegrinelli Jean-Marie, Daelemans Caroline

**Affiliations:** ^1^ Obstetrics Division/Department of Woman, Child and Adolescent, Geneva University Hospitals, Geneva, Geneva State, Switzerland, hug-ge.ch; ^2^ Department of Pediatrics, Gynecology, and Obstetrics, Geneva University Hospitals and University of Geneva, Geneva, Geneva State, Switzerland, unige.ch; ^3^ Gynecology Division/Department of Woman, Child and Adolescent, Geneva University Hospitals, Geneva, Geneva State, Switzerland, hug-ge.ch

**Keywords:** case report, cervical oedema, cervical trauma, multiparous woman, pushing before full dilation

## Abstract

**Introduction:**

Cervical oedema during labour is relatively common, but severe circumferential oedema persisting at advanced dilation is rare, particularly in multiparous women. In such cases, the swollen cervix may mimic an obstructive mass and raise concern for labour obstruction, cervical laceration or avulsion if pushing is initiated.

**Case Presentation:**

We report the case of a 41‐year‐old Gravida 5, Para 4 woman who underwent induction of labour at 39 weeks′ gestation for diet‐controlled gestational diabetes. Labour progressed to 8 cm dilation, at which point a tense, circumferential, purple‐hued cervical swelling developed. The clinical presentation raised concern for obstructed labour and potential cervical trauma, although maternal and fetal parameters remained reassuring. The main diagnostic challenge was to distinguish severe reactive cervical oedema from cervical prolapse, haematoma or other cervical pathology. After interdisciplinary discussion and detailed counselling, the patient chose to proceed with a cautious trial of pushing. This decision was based on multiparity, a deeply engaged fetal head, reassuring fetal status, the absence of clinical obstruction to descent and the immediate availability of senior obstetric support and emergency resources. She achieved spontaneous vaginal delivery of a healthy infant within two pushes, without instrumentation, cervical trauma, postpartum haemorrhage or neonatal complication. The cervix remained oedematous but intact. Partial resolution was observed by postpartum Day 3, and follow‐up colposcopy at 2 months showed no suspicious cervical lesion. Written informed consent for publication was obtained from the patient.

**Discussion:**

This case illustrates that, in carefully selected multiparous women, severe reactive cervical oedema at advanced dilation may not necessarily preclude vaginal birth. However, this observation is hypothesis generating and should not be generalised to nulliparous women, cases with high fetal station, suspected obstruction, fetal compromise or limited access to emergency obstetric care.

## 1. Introduction

Although moderate cervical oedema during labour is relatively common, significant localised oedema persisting at near‐complete dilation is rare, particularly in multiparous women, and may pose a clinical dilemma. In such cases, reactive cervical swelling may appear clinically as an obstructive mass, raising concern for potential cervical laceration or avulsion if pushing is initiated, even when the underlying process is benign and labour‐related. We present a case where severe cervical oedema during labour led to apparent arrest of dilation and raised concerns regarding the safety of vaginal delivery. This case highlights the value of clinical judgement, interdisciplinary collaboration and shared decision‐making in complex scenarios, particularly when the diagnosis remains uncertain and intervention carries significant risk.

To the authors′ knowledge, a focused PubMed search using combinations of the terms ‘cervical oedema,’ ‘cervical edema,’ ‘labour,’ ‘labor,’ ‘vaginal delivery,’ ‘vaginal birth,’ ‘cervical prolapse’ and ‘cervical haematoma’ identified no previous report describing successful vaginal birth despite severe circumferential reactive cervical oedema at advanced dilation, without true cervical prolapse, obstructed fetal descent or cervical trauma.

## 2. Case Report

A 41‐year‐old Gravida 5, Para 4 woman presented at 39 weeks′ gestation to our tertiary hospital for induction of labour due to diet‐controlled gestational diabetes. Her obstetric history included four previous uncomplicated spontaneous vaginal deliveries at outside institutions. This pregnancy was unplanned but progressed uneventfully.

On admission, cervical examination revealed a posterior cervix, approximately 2 cm dilated and 30%–40% effaced. Artificial rupture of membranes (AROM) was initially not feasible, as the membranes were closely applied to the fetal head; therefore, intravenous oxytocin was initiated in accordance with institutional protocol. After 2 h of contractions, the cervix had softened, become anterior and accessible, allowing for AROM. After 3 h, the cervix was 4 cm dilated and fully effaced, with a thin anterior cervical lip noted at the 10 o′clock position.

Table 1 summarises the main clinical milestones.

**Table 1:** Timeline of labour and postpartum follow‐up.

Time from oxytocin initiation	Clock time	Clinical event
0 h	14:00	Oxytocin induction started after unsuccessful artificial rupture of membranes (AROM). Cervix two fingers dilated, posterior, moderately soft; membranes closely applied to the fetal head; cephalic presentation high at −3 cm below the ischial spines.
+2 h and 30 min	16:30	AROM performed, with abundant clear amniotic fluid.
+9 h and 30 min	23:30	Cervix 4 cm dilated and fully effaced; thin anterior cervical lip noted at 10 o′clock.
+10 h and 45 min	00:45	Cervix 6 cm dilated.
+12 h and 50 min	02:50	Severe circumferential cervical oedema became apparent; cervix approximately 8 cm dilated.
+17 h	07:00	Persistent circumferential cervical oedema; cervix remained approximately 8 cm dilated.
+18 h and 20 min	08:20	Interdisciplinary discussion and initiation of cautious pushing.
+18 h and 38 min	08:38	Spontaneous vaginal delivery.
Postpartum Day 3	—	Partial resolution of cervical oedema.
Two months postpartum	—	Colposcopy showing residual physiological cervical alteration without suspicious lesion.

Labour continued with descent of the fetal head. However, the previously thin cervical lip became increasingly oedematous, eventually expanding circumferentially into a tense, purple‐hued oedematous swelling occupying the upper vaginal canal. Cervical dilation appeared to stall at approximately 8 cm, with complete effacement except for the protruding oedematous tissue. The mass was tense and nonreducible, raising concern for obstructed labour, possible cervical trauma and diagnostic uncertainty regarding its aetiology.

Repeated intrapartum examinations, progression from a thin cervical lip to circumferential oedematous swelling during labour in the absence of prior findings, and postpartum regression of the lesion supported a diagnosis of labour‐related reactive cervical oedema. The main diagnostic challenge was differentiating this condition from cervical prolapse, haematoma and, more rarely, neoplastic pathology (Table 2).

**Table 2:** Diagnostic reasoning and differential diagnosis.

Differential diagnosis	Findings supporting/arguing against diagnosis
Reactive cervical oedema	Rapid development during labour; prior thin cervical lip; progression with fetal descent; tense nonindurated tissue; postpartum regression
Cervical prolapse	Considered, but no separate prolapsing cervix or uterovaginal descent; swelling remained labour‐related and fetal descent continued
Cervical haematoma	Considered, but no unilateral expanding mass, fluctuance, active bleeding or haemodynamic instability
Neoplastic cervical lesion	Considered unlikely because of rapid intrapartum onset, absence of prior lesion and reassuring postpartum colposcopy

**Figure 1:**
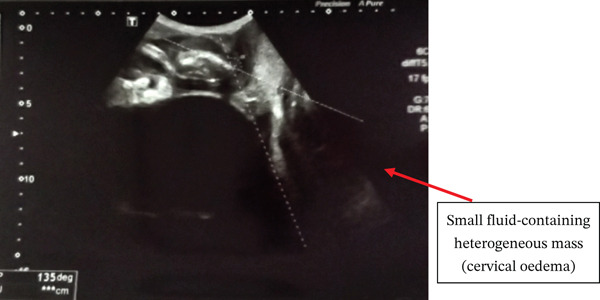
Intrapartum ultrasound showing fetal head engagement.

An intrapartum ultrasound confirmed fetal head engagement but was of limited value in characterising the cervical mass, which appeared as a small fluid‐containing heterogeneous structure (Figure 1). The rapid intrapartum development of the lesion, its tense but nonindurated consistency, and the absence of prior cervical abnormalities supported a diagnosis of labour‐related oedema. The swelling was circumferential but remained continuous with the cervix; there was no separate prolapsing organ, no eversion suggestive of true uterovaginal prolapse and fetal descent was maintained.

A cervical haematoma was considered possible because of the purple discoloration, progressive enlargement and the tense appearance of the tissue. However, this diagnosis was considered less likely because there was no unilateral expanding collection, no active bleeding and no maternal haemodynamic instability. Neoplastic pathology was considered unlikely because no cervical lesion had been identified before labour, the change developed rapidly during labour and the tissue appeared oedematous rather than indurated or friable.

At this stage, the labour met criteria for arrest of dilation, and caesarean section was being actively considered by the team. However, given that the fetal head remained deeply engaged, the patient was multiparous, and maternal as well as fetal parameters were reassuring, the incoming team was consulted for a second opinion.

**Figure 2:**
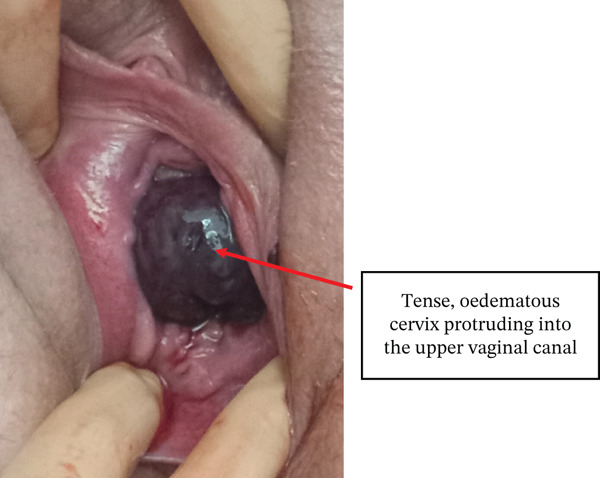
Severe circumferential reactive cervical oedema during labour.

A photo was taken at the onset of active pushing, highlighting the striking appearance of the cervical mass (Figure 2).

The patient was counselled regarding the risks of cervical laceration or avulsion, and was informed that, if the lesion corresponded to a haematoma, pushing could increase the risk of bleeding. Considering the overall clinical context, the team offered a trial of cautious, controlled pushing to avoid caesarean delivery, with the understanding that cervical trauma could occur, potentially increasing the risk of haemorrhage or complications in future pregnancies. The clinical criteria supporting this decision were explicitly reviewed: multiparity with four previous vaginal births, completion of the patient′s family plans, deeply engaged fetal head, reassuring fetal heart rate, absence of maternal instability, no evidence that the oedematous cervix was preventing fetal descent and immediate access to senior obstetric staff, operating theatre, anaesthesia and blood products. The plan was to stop pushing immediately in case of bleeding, tissue tearing, loss of fetal descent, worsening maternal condition or nonreassuring fetal status. Emergency operative delivery or caesarean section remained available at any time. After reflection, the patient opted to attempt a vaginal delivery, explicitly stating that she had completed her family and accepted the associated risks. This case underscores the importance of patient autonomy, individualised delivery planning and shared decision‐making in situations with uncertain outcomes. Informed consent for publication of the case details and clinical images was obtained from the patient.

Given the incomplete dilation and oedema, vacuum extraction was preferred over forceps should instrumentation become necessary, and two units of red blood cells were ordered pre‐emptively.

**Figure 3:**
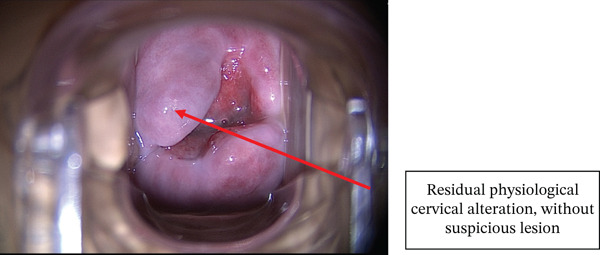
Colposcopic examination at 2 months postpartum.

The patient subsequently achieved spontaneous vaginal delivery of a healthy female infant within two pushes, with an intact perineum. The neonate weighed 3430 g and adapted well (Apgar 9/10/10, umbilical vein pH 7.26). The patient tolerated the management approach well, and no complications related to the trial of pushing or vaginal delivery were observed. Immediate postpartum examination revealed a markedly oedematous but intact cervix. By postpartum Day 3, the oedema had partially resolved, with some residual swelling remaining. Postpartum recovery was uneventful. Colposcopic follow‐up at 2 months postpartum demonstrated a residual cervical alteration at the 10 o′clock position, considered consistent with a normal multiparous cervix and not suspicious for malignancy (Figure 3).

Informed consent for publication of case details and clinical images was obtained from the patient.

## 3. Discussion

This case illustrates a rare presentation of severe circumferential reactive cervical oedema at advanced dilation, likely resulting from venous and lymphatic obstruction of a persistent cervical lip compressed between the descending fetal head and the maternal pelvis. This mechanism may have been amplified by multiparity, rapid fetal descent and an early urge to push before complete dilation, explaining the progression from a thin anterior cervical lip to severe circumferential oedema [1–3]. Some authors have suggested that, in selected multiparous patients with a soft cervix, allowing pushing despite incomplete dilation may facilitate delivery without adverse outcomes [3].

Reported management strategies vary according to the appearance, reducibility and presumed cause of the swelling [1, 2, 4–8]. In some reports, cervical oedema improved with conservative measures such as maternal repositioning and local cooling [1]. Pharmacological approaches have also been described, including diphenhydramine and hyoscine butylbromide [1, 9]. In contrast, true cervical or uterovaginal prolapse, obstructed descent or cervical dystocia have required operative management, including caesarean section or cervical incisions [4–8]. The present case differed from these scenarios because the swelling was severe and nonreducible, but fetal descent continued and there was no evidence of true prolapse or fixed mechanical obstruction. Partial postpartum resolution further supported the benign and self‐limited nature of the condition.

Importantly, this case should not be interpreted as an endorsement of pushing before full dilation. The approach was highly individualised and was undertaken only because favourable clinical conditions and immediate operative capability were present. The ethical balance between maternal autonomy and provider responsibility was central to management. The patient wished to avoid caesarean delivery if safely possible and explicitly accepted the risk of cervical trauma after counselling. However, the team retained responsibility for limiting risk by defining clear criteria for abandoning the trial of pushing if maternal or fetal safety became compromised. The team also recognised that decision‐making during active labour may be influenced by pain, fatigue and emotional distress, and therefore ensured repeated confirmation of the patient′s understanding throughout the decision‐making process.

Cervical trauma was the main anticipated complication. Cervical lacerations after vaginal birth are uncommon but may be associated with postpartum haemorrhage, need for surgical repair and possible implications for future pregnancies [10, 11]. In this case, the risk was considered potentially increased because pushing was initiated before complete dilation and because the cervix was tense and oedematous. This risk was discussed explicitly with the patient. The contingency plan included immediate reassessment of the cervix after each push if needed, discontinuation of pushing in case of bleeding or tissue tearing, readiness for operative delivery or caesarean section and pre‐emptive availability of blood products.

Finally, it is important to recognise that, in multiparous women, such presentations may unmask underlying pelvic floor weakness or early uterovaginal prolapse [4–6]. Postpartum follow‐up with colposcopic and pelvic floor assessment is therefore recommended to ensure resolution and to evaluate cervical and pelvic floor structural integrity. Longer term follow‐up may be useful when feasible, particularly to assess persistent cervical anatomical changes, cervical competence in a subsequent pregnancy if relevant, pelvic floor symptoms, abnormal bleeding, dyspareunia and signs of prolapse.

This report has several limitations. As a single case, the findings cannot be generalized, and the favourable outcome observed may partly reflect the patient′s multiparity, favourable fetal station and reassuring maternal–fetal status. Further observations would be needed to determine whether this approach can be safely applied in similar situations. This case should not be extrapolated to nulliparous women, cases with high fetal station, suspected true obstruction, macrosomia, nonreassuring fetal status, active bleeding or settings where emergency operative delivery is not immediately available. The follow‐up period was also limited to 2 months, which may not capture all potential long‐term cervical or pelvic floor consequences. Therefore, this report should be interpreted as a hypothesis‐generating observation rather than evidence supporting routine pushing before full dilation.

## 4. Conclusion

Severe cervical oedema can mimic an obstructive mass and complicate labour management. In carefully selected multiparous women with reassuring maternal and fetal status, a cautious trial of vaginal delivery may be safe. Postpartum follow‐up remains essential to ensure resolution and exclude underlying pathology.

**Nomenclature**
AROM: artificial rupture of membranes

## Author Contributions

All authors contributed to the clinical interpretation of the case and to the preparation or critical revision of the manuscript. Dr. Pécheux Océane corresponding author and manuscript guarantor, had full access to all of the data in this case report and takes complete responsibility for the integrity of the data and the accuracy of the data analysis. Pécheux Océane and Patet Emma are co first authors

## Funding

No funding was received for this manuscript. Open access publishing facilitated by Universite de Geneve, as part of the Wiley ‐ Universite de Geneve agreement via the Consortium Of Swiss Academic Libraries.

## Disclosure

Dr. Pécheux Océane, corresponding author and manuscript guarantor, affirms that this manuscript is an honest, accurate and transparent account of the case being reported; that no important aspects of the case have been omitted; and that any discrepancies from the case as managed and reported have been explained. All authors have read and approved the final version of the manuscript. The corresponding author is the guarantor of submission.

## Consent

Informed consent for publication of case details and clinical images was obtained from the patient.

## Conflicts of Interest

The authors declare no conflicts of interest.

## Data Availability

The data supporting the findings of this case report are included within the article. No additional datasets were generated or analysed during the current study. Further clinical details cannot be shared publicly to protect patient confidentiality.

## References

[1] LoGiudice J. A. , Holland E. , and Esposito C. P. , Midwifery Management of a Birthing Person With Cervical Edema During Labor, Journal of Midwifery & Women′s Health. (2022) 67, no. 5, 644–650, 10.1111/jmwh.13406, 36215142. 36215142

[2] Joshi L. , Obstructed Labour Due to Oedematous Cervical Lip, Journal of Obstetrics and Gynecology of India. (1971) 21, no. 2, 633–634.

[3] Ferguson R. , Early Pushing Urge in the Laboring Patient, Nursing. (2018) 48, no. 2, 69–71, 10.1097/01.NURSE.0000529819.51901.8a, 29369283. 29369283

[4] Worku A. B. , Kebede M. A. , Wudineh A. A. , Techane A. G. , Lakew M. D. , and Zeleke C. A. , Cervical Prolapse During Labor: A Case Report, Case Reports in Women′s Health. (2025) 45, e00690, 10.1016/j.crwh.2025.e00690, 39974582. PMC11836502 39974582

[5] Maki J. , Mitoma T. , Mishima S. , Ohira A. , Tani K. , Eto E. , Hayata K. , and Masuyama H. , A Case Report of Successful Vaginal Delivery in a Patient With Severe Uterine Prolapse and a Review of the Healing Process of a Cervical Incision, Case Reports in Women′s Health. (2022) 33, e00375, 10.1016/j.crwh.2021.e00375, 34987980. PMC8703083 34987980

[6] Han K. H. , Shin J. J. , Shin M. S. , Kim B. J. , Hwang K. R. , Jun H. W. , and Bae K. B. , A Third Stage Pelvic Organ Prolapse due to Cervical Swelling During Labor: A Case Report, Korean Journal of Obstetrics & Gynecology. (2010) 53, no. 8, 727–731, 10.5468/kjog.2010.53.8.727.

[7] Neri A. , Ovadia Y. , Schoenfeld A. , and Nitke S. , Detachment of Posterior Uterine Cervical Lip Associated With Spontaneous Delivery, European Journal of Obstetrics and Gynecology and Reproductive Biology. (1982) 13, no. 5, 291–292, 10.1016/0028-2243(82)90051-x, 7117660. 7117660

[8] Verma M. L. , Tripathi V. , Singh U. , and Rahman Z. , Salvage From Cervical Dystocia in Third Degree Uterovaginal Prolapse: Duhrssen′s Incision, BMJ Case Reports. (2018) 2018, bcr2017223821, 10.1136/bcr-2017-223821, 29444799. PMC5847846 29444799

[9] Al Qahtani N. H. , The Effect of Hyoscine Butylbromide in Shortening the First Stage of Labor: A Double Blind, Randomized, Controlled Clinical Trial, Therapeutics and Clinical Risk Management. (2011) 7, 495–500, 10.2147/TCRM.S16480. 22241946 PMC3253756

[10] Melamed N. , Ben-Haroush A. , Chen R. , Kaplan B. , and Yogev Y. , Intrapartum Cervical Lacerations: Characteristics, Risk Factors, and Effects on Subsequent Pregnancies, American Journal of Obstetrics & Gynecologys. (2009) 200, no. 4, 388.e1–388.e4, 10.1016/j.ajog.2008.10.034, 19200938. 19200938

[11] Wong L. F. , Wilkes J. , Korgenski K. , Varner M. W. , and Manuck T. A. , Intrapartum Cervical Laceration and Subsequent Pregnancy Outcomes, AJP Reports. (2016) 6, no. 3, e318–e323, 10.1055/s-0036-1592198, 27621953. 27621953 PMC5017884

